# Analysis of Subset Chimerism for MRD-Detection and Pre-Emptive Treatment in AML

**DOI:** 10.3389/fonc.2022.841608

**Published:** 2022-02-17

**Authors:** Julia-Annabell Georgi, Sebastian Stasik, Martin Bornhäuser, Uwe Platzbecker, Christian Thiede

**Affiliations:** ^1^ Medizinische Klinik und Poliklinik I, Universitätsklinikum Carl Gustav Carus der Technischen Universität, Dresden, Germany; ^2^ National Center for Tumor Diseases (NCT), Dresden, Germany; ^3^ Klinik und Poliklinik für Hämatologie, Zelltherapie und Hämostaseologie, Universitätsklinikum Leipzig, Leipzig, Germany; ^4^ AgenDix GmbH, Dresden, Germany

**Keywords:** AML, allogeneic cell transplantation, relapse, detection, subset chimerism, pre-emptive treatment

## Abstract

Allogeneic hematopoietic stem cell transplantation (alloHCT) represents the only potentially curative treatment in high-risk AML patients, but up to 40% of patients suffer from relapse after alloHCT. Treatment of overt relapse poses a major therapeutic challenge and long-term disease control is achieved only in a minority of patients. In order to avoid post-allograft relapse, maintenance as well as pre-emptive therapy strategies based on MRD-detection have been used. A prerequisite for the implementation of pre-emptive therapy is the accurate identification of patients at risk for imminent relapse. Detection of measurable residual disease (MRD) represents an effective tool for early relapse prediction in the post-transplant setting. However, using established MRD methods such as multicolor flow cytometry or quantitative PCR, sensitive MRD monitoring is only applicable in about half of the patients with AML and advanced MDS undergoing alloHCT. Donor chimerism analysis, in particular when performed on enriched leukemic stem and progenitor cells, e.g. CD34+ cells, is a sensitive method and has emerged as an alternative option in the post alloHCT setting. In this review, we will focus on the current strategies for lineage specific chimerism analysis, results of pre-emptive treatment using this technology as well as future developments in this field.

## Introduction

Acute myeloid leukemia (AML) describes a group of hematological malignancies originating from hematopoietic stem and progenitor cells. Despite major advances in the understanding of disease mechanisms over the last decades, which eventually led to improvements in therapy and targeted treatment options in subgroups of patients, outcome of affected individuals is still suboptimal, and the majority of AML patients will eventually succumb to their disease. Allogeneic hematopoietic stem cell transplantation (alloHCT), first successfully performed more than 50 years ago (1), still represents the only curative option, with AML currently being the most common indication for alloHCT worldwide (2). However, even after this intensive treatment, substituting the entire hematopoietic system, leukemic stem cells can survive and lead to disease recurrence, with up to 40% of patients suffering from relapse (3).

Treatment of recurrent disease after alloHCT remains a clinical challenge, associated with dismal prognosis and long-term disease-free survival between 10-20% (4). Thus, strategies to avoid overt relapse have been a major field of research during the last decades (5, 6). Several studies have shown that treating disease recurrence already at a subclinical state, so called “minimal”, or more recently re-termed “measurable residual disease” (MRD) stage, is associated with substantially improved outcome (7). Measurement of MRD in AML is more complex than in other diseases, such as ALL or CML, because there is no common target, implicating technical challenges in the choice of the most appropriate method, selection of cut-offs for intervention as well as in the standardization of procedures (8). Nevertheless, several markers have been successfully used for MRD-detection post alloHCT, including recurrent translocations such as RUNX1::RUNX1T1, CBFb::MYH11, or NUP214::CAN, multicolor flow cytometry (MFC), aberrant expression of the WT1-gene and more recently, next generation sequencing (NGS) (9). However, besides these methods also in use in the general AML population, in patients after allogeneic stem cell transplantation, detection of recipient cells based on the different DNA profiles between donor and recipient, i.e. the detection of chimerism, represents an alternative method for assessment of MRD. This review will briefly summarize current therapeutic strategies for relapse in patients with AML after alloHCT, and then focus specifically on the use of chimerism analysis in sorted stem and progenitor cells for MRD detection, treatment initiation and monitoring in AML patients post alloHCT.

## Relapse After Allogeneic Stem Cell Transplantation

Besides the toxicity associated with the procedure, relapse of leukemia remains the single most important cause of death after allogeneic stem cell transplantation in patients with AML (4). The incidence of relapse is associated with several factors, including patient age, cytogenetic and molecular risk factors, intensity of the conditioning regimen, the disease stage (CR1 or >CR1) as well as the MRD-status prior to transplantation (10). In a recent large analysis involving 2289 patients with AML performed by the Center for International Blood and Marrow Transplant Research (CIBMTR), the risk of relapse at 2 years was 37.5% in patients within the ELN-adverse cytogenetic group, going up to 45% in patients with monosomies of chromosomes 5 or 7, but still reaching 28% in favorable or intermediate risk patients (11).

Treatment of hematological relapse after alloHCT is associated with only moderate response. In a retrospective analysis performed by Thanarajasingam et al., reduction of immunosuppression did not impact outcome after relapse, whereas the receipt of donor lymphocyte infusion (DLI) or a second HCT was associated with an improved outcome at 31% overall survival (OS) at 3 years compared to 13% in patients not treated with DLI or second HCT (12). Patients receiving chemotherapy only had a 3-year OS of 19%. Even novel agents are not associated with substantially improved outcome, e.g. in the ADMIRAL study, treatment with the FLT3-inhibitor Gilteritinib as single agent resulted in a CR rate of 35.4% in patients relapsing after HCT, with a median OS of 8.3 months (13).

### Prophylactic vs. Pre-Emptive Treatment

Given the persistently high relapse rates in AML patients after alloHCT and the limited number of curative options once overt relapse has occurred, the need for treatment strategies preventing or deferring disease recurrence is immanent. One potential strategy for maintaining disease control in the post-transplant setting is the administration of maintenance or prophylactic therapy. To increase the graft vs leukemia (GvL) effect, several studies used prophylactic treatment with DLI post alloHCT. Schmid et al. showed that prophylactic DLI given to a subset of 12 high-risk AML patients after FLAMSA-RIC based alloHCT induced sustained remission in 10 patients, indicating the potency of the GvL effect in disease control (14). These promising results were confirmed in subsequent studies (15, 16). However, especially in patients with intermediate risk cytogenetics, the curative potential of donor T-cells has to be weighed against the potential sequelae of graft-versus-host-disease (GvHD) associated with this treatment.

The agent most extensively employed for maintenance therapy is 5-azacytidine (azacitidine; AZA). Oran and colleagues very recently reported the results of the first phase III randomized controlled trial investigating the efficacy and safety of azacitidine maintenance in the post-transplant setting. In this study, 187 patients with high-risk AML or MDS who were in CR after alloHCT received AZA or placebo at a dose of 32 mg/m^2^ on day 1 to 5 for up to 12 cycles. Despite the encouraging results of phase II studies examining hypomethylating agents as a maintenance strategy after HCT (17–20), this trial reported no improvement in relapse-free survival, with a median of 2.07 years in the azacytidine group vs 1.28 years in the control group (p= .43).

In contrast, molecularly targeted maintenance treatment appears to offer better disease control. In the placebo-controlled SORMAIN trial, Burchert and coworkers were able to show that maintenance therapy with sorafenib significantly improves outcome after alloHCT in FLT3-ITD-mutated AML with a 2-year relapse-free survival (RFS) of 85% in the sorafenib group vs 53.3% in the placebo group (p= .002) (21). In addition to sorafenib, midostaurin is another FLT3 inhibitor approved for upfront treatment together with intensive chemotherapy (22), which is currently under investigation for use in maintenance therapy after alloHCT. The RADIUS trial was the first randomized trial to investigate the efficacy of maintenance therapy with midostaurin after alloHCT in *FLT3*-ITD-positive AML. The study was not powered to detect a treatment difference, yet there was a trend towards a better outcome with midostaurin with a 13% improvement in 1.5-year RFS (23). These results support those reported by Schlenk and colleagues, showing an improved event-free survival (EFS) and OS in patients with *FLT3*-ITD-positive AML starting maintenance therapy with midostaurin within 100 days post-transplant compared to patients having received midostaurin in induction and consolidation only (24).

Based on the encouraging results of targeted maintenance strategies based on FLT3 inhibition, IDH inhibitors are another promising substance group for prevention of relapse after alloHCT. Two ongoing Phase I/II trials are currently evaluating the safety and preliminary efficacy of maintenance therapy with enasidenib in *IDH2*-mutated myeloid neoplasms after alloHCT (ClinicalTrials.gov identifiers NCT03515512, NCT04522895).

Nevertheless, patients with targetable mutations such as *FLT3*, *IDH1* or *IDH2* represent only about 20-30% of patients transplanted, so for the vast majority of individuals, effective treatment options are still lacking. Thus, although available data on post-transplant maintenance therapy clearly merit further investigation, these prophylactic regimens have not yet shown the anticipated therapeutical benefit.

A competing concept aiming at the prevention of overt hematological relapse in the post-transplant setting is pre-emptive therapeutic intervention on the appearance of measurable residual disease (MRD). A prerequisite for the implementation of pre-emptive therapy is the accurate identification of patients at risk for imminent relapse. Given the limited availability of suitable MRD-markers amenable for high sensitivity tracing of leukemic recurrence, especially in high-risk patients undergoing alloHCT, the assessment of chimerism has been used as an alternative approach.

### Analysis of Subset Chimerism

After alloHCT, the recurrence of recipient-derived hematopoiesis has been shown to be associated with an increased risk of relapse (5, 25–27). Analysis of DCC has become a basic diagnostic requirement for monitoring AML patients after alloHCT and has been extensively studied as a surrogate for immanent relapse.

One limitation of chimerism analysis using unsorted material for MRD assessment is that the method does not differentiate between non-malignant cells (e.g. T-cells) and cells of the leukemic clone, thus mixed chimerism per se does not necessarily herald relapse. However, several groups have shown that increasing mixed chimerism post alloHCT is associated with an increased risk of relapse (28).

**Figure 1:**
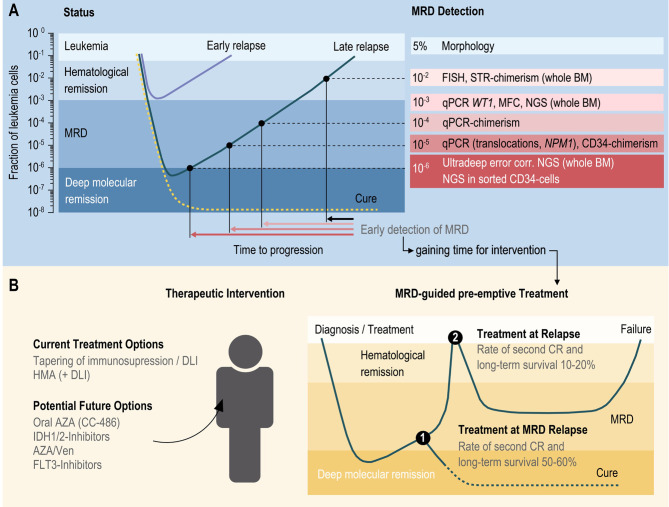
Schematic illustration of MRD detection **(A)** and treatment options of AML patients following allogeneic HCT **(B)**. MRD measurable residual disease; FISH fluorescence in situ hybridization; STR short tandem repeat; BM bone marrow; qPCR quantitative polymerase chain reaction; WT1 Wilms tumor protein 1; MFC multicolor flow cytometry immunophenotyping; NGS next generation sequencing; NPM1 Nucleophosmin 1; HMA hypomethylating agents; DLI donor lymphocyte infusion; AZA azacytidine; Ven Venetoclax; CR complete remission; IDH Isocitrate dehydrogenase; FLT3 Fms related receptor tyrosine kinase 3.

As another drawback, despite significant technical advances in the field of chimerism analysis over the past decades, analysis of total donor cell chimerism is still compromised by a limited level of sensitivity, ranging between 1-5% for STR (short tandem repeats)-based approaches on whole peripheral blood (PBL) or bone marrow (BM) (29) down to 0.1% using quantitative real-time PCR for single nucleotide variants (SNVs) (28, 30, 31) or digital-PCR for In/Del-polymorphisms (32). Consequently, the interval between the detection of the decrease in donor cell chimerism and the clinical diagnosis of relapse is often too short to enable successful pre-emptive therapeutic intervention (illustrated in **Figure 1A**).

More sensitive detection of residual or recurrent leukemic cells can be achieved by monitoring the lineage specific chimerism, which has been demonstrated to be a highly sensitive and specific surrogate for impending relapse. The concept of chimerism analysis of cellular subsets after alloHCT dates back to a 1985 study by Ginsberg and colleagues (33). CD34, a marker of normal early stem and progenitor cells, is also expressed on blast cells in more than 70-80% of all AML cases (34). Major improvements in selection technologies (i.e. immunomagnetic enrichment using paramagnetic beads, high throughput cell sorters) as well as the implementation of molecular tools capable of using minute amounts of DNA (i.e. multiplex STR-PCR and qPCR) have greatly facilitated this work. As aggregated in **Table 1**, numerous groups have reported on the use of CD34, CD33 or CD117 for the enrichment of early myeloid progenitors for MRD detection.

**Table 1:** Selection of published data on MRD detection with subset chimerism performed in AML or MDS patients following allogeneic HCT.

Study	Study design	Lineage specific chimerism	Study population monitored by LSC	Method	Cutoff level LSC	Time from LSC decrease to hematologic relapse	Intervention
Mattsson et al. (35)	prospective	CD33+ and CD13+ DCC in BM and PBL	30 patients (22 AML, 6 MDS, 1 CMML, 1 BAL)	MACS pre-enrichment and subsequent FACS, VNTR-PCR	semi-quantitative analysis	median 66 days (range 23–332)	none
Scheffold et al. (36)	prospective	CD34+ DCC in BM	20 patients with AML	MACS pre-enrichment and subsequent FACS, STR-PCR	<75%	21-91 days	*Relapse treatment:* DLI, low-dose ARA-C 2×10 mg/m^2^ daily for 14 days, GM-CSF 75 μg/m^2^ daily for 4 weeks
Zeiser et al. (37)	prospective	CD34+ DCC in BM and PBL	168 patients (137 AML, 31 MDS)	MACS, STR-PCR	>5% decrease	at least 10 days	*Pre-emptive therapy:* rapid tapering of systemic immunosuppression or DLI
Bornhäuser et al. (38)	prospective	CD34+ DCC in BM and PBL	90 patients (67 AML, 7 MDS, 16 ALL)	MACS pre-enrichment and subsequent FACS, STR-PCR	<80%	median 61 days (range 0-567)	*Pre-emptive therapy:* rapid tapering of systemic immunosuppression or DLI
Sairafi et al. (39)	retrospective	CD3+, CD19+, CD33+ DCC in BM	118 patients (29 AML, 14 MDS, 39 CML, 24 ALL, 12 others)	MACS pre-enrichment and subsequent FACS, VNTR-PCR	not specified	–	*Prophylactic or pre-emptive treatment:* DLI
Lange et al. (29)	retrospective	CD34+ DCC in BM	88 patients (68 AML, 20 MDS)	FACS, STR-PCR	<90% or >5% decrease	–	none
Platzbecker et al. (40)	prospective	CD34+ DCC in PBL	20 patients with AML/MDS	MACS pre-enrichment and subsequent FACS, STR-PCR	<80%	–	*Pre-emptive treatment:* AZA 75 mg/m^2^ on days 1-7 for up to 4 cycles
Rosenow et al. (27)	retrospective nested case control study	CD34+ DCC in BM	134 patients (126 AML, 8 MDS)	MACS pre-enrichment and subsequent FACS, STR-PCR	<90%	median 56 days (range: 14–546)	*Pre-emptive treatment:* rapid tapering of systemic immunosuppression and subsequent DLI
Hoffmann et al. (41)	prospective	CD34+ DCC in PBL	85 patients with AML/MDS	semi-automated enrichment MACS, STR-PCR	<80%	29–42 days	none
Platzbecker et al. (42)	prospective	CD34+ and CD117+ DCC in PBL	107 patients with AML/MDS	MACS pre-enrichment and subsequent FACS, STR-PCR	<80%	–	*Pre-emptive treatment:* AZA 75 mg/m^2^ on days 1–7 for up to 24 cycles
Guillaume et al. (43)	retrospective	CD34+ DCC in BM	52 patients (34 AML, 18 MDS)	MACS pre-enrichment and subsequent FACS, STR-PCR	<95%	in retrospective analysis mixed CD34+ DCC did not correlate with relapse	*Maintenance therapy:* AZA 32 mg/m^2^ on days 1-5 for up to 12 cycles + DLI for up to 3 cycles

DCC, donor cell chimerism; LSC, lineage specific chimerism; BM, bone marrow; PBS, peripheral blood leukocytes; AML, acute myeloid leukemia; MDS, myelodysplastic syndrome; CMML, chronic myelomonocytic leukemia; BAL, biphenotypic acute leukemia; MACS, magnetic activated cell sorting; FACS, fluorescence activated cell sorting; VNTR, variable number of tandem repeats; STR, short tandem repeat; PCR, polymerase chain reaction; DLI, donor lymphocyte infusion; AZA, azacytidine.

After first studies reporting individual cases (44) or using qualitative methods (35) indicated the principal feasibility of MRD detection using lineage specific chimerism, Scheffold and colleagues first published evidence of the predictive value of CD34 lineage specific chimerism in AML in 2004. In a cohort of 20 AML patients the authors demonstrated that a decrease in bone marrow CD34+ DCC to below 75% was highly predictive of relapse in patients with a CD34+ leukemic phenotype (36). CD34+ DCC decreased 21-91 days before diagnosis of hematological relapse. In contrast, chimerism analyses of T cells, B cells and monocytes were not or far less sensitive for detection of impending relapse. In 2009, we published a prospective analysis on the feasibility of CD34 lineage specific chimerism assessment in PBL and BM samples to monitor MRD in 90 patients with AML or MDS after alloHCT (38). The study confirmed that decreasing CD34+ DCC is an independent predictor of relapse and inferior survival. Mixed or decreasing subset DCC was documented in 28 (80%) patients before they experienced relapse. The median interval between a decrease of CD34+ DCC < 80% and hematological relapse was 61 days, with MRD detected in some patients up to 567 days before overt relapse. In contrast, a decrease in total donor cell chimerism was documented a median of only nine days before hematological relapse. The sensitivity of the method in patients with CD34+ leukemia was shown to be comparable to that of PCR assays for the amplification of leukemia-specific transcripts or mutated DNA sequences (38, 44). Additional validation experiments performed on this method using cell line dilutions demonstrate a sensitivity down to 1x10^-5^ to 5x10^-6^, further illustrating a very high level of sensitivity of this procedure (unpublished data).

In a complementary report by Rosenow and colleagues monitoring of CD34-lineage specific chimerism in BM was confirmed as a highly sensitive and specific diagnostic tool for the identification of AML and MDS patients at risk for hematological relapse after alloHCT (27). In this retrospective case control study, lineage-specific DCC were measured in 126 patients with AML and 8 with MDS. Impending relapse with an incomplete CD34+ subset DCC <90% was detected in 43 patients. The median time from diagnosis of decreasing subset DCC to consecutive relapse was 56 days. Patients with a stable CD34+ DCC showed a significantly better RFS after 3 years with 74% as compared to 40% in patients with decreasing CD34+ DCC.

In conclusion, CD34+ lineage-specific chimerism analysis for monitoring AML patients after alloHCT is a feasible and sensitive tool for early MRD detection and relapse prediction. Recent efforts to enable semi-automated detection may further facilitate the use of CD34-specific chimerism. Hoffmann and colleagues screened 85 patients for CD34+ DCC using a semi-automated analysis procedure without the need for flow cytometric cell sorting (41). A loss of CD34+ DCC to <80% invariably predicted subsequent hematological recurrence. A significant decrease in CD34+ DCC was detected 29-42 days before overt hematological relapse, thus slightly later than the median of 50-60 days reported procedures involving flow-sorting (27, 36, 38).

Summarizing these data, most studies were able to document that increasing mixed chimerism in myeloid progenitors allows significantly earlier detection of relapse compared to analyses of unselected material. So far, the majority of groups focused on the use of bone marrow for stem cell enrichment (27, 29, 36). However, it is known for long-time that CD34+ cells are also detectable in the steady-state hematopoiesis (45). Kato and Radbruch first employed a combination of magnet-activated cell sorting (MACS) and fluorescent activated cell sorting (FACS) to facilitate enrichment of these cells in PBL samples, enriching them from 0.18% (+/- 0.052%) to more than 98% (46). Using peripheral blood as starting material has several advantages, most importantly that monitoring can be performed in shorter intervals. In most studies summarized in **Table 1**, CD34-selection from PBL was associated with longer intervals between MRD detection and subsequent hematological relapse, the median interval was in the range of two months (27, 35, 38, 41). In line with this, we were able to show that in pairwise analyses of samples taken at the same time, the level of residual recipient cells in PBL-CD34+ cells was significantly higher compared to matched BM-derived CD34+ cells (38).

## Use of Subset-Chimerism to Guide Pre-Emptive Treatment

Several studies have shown that this early detection of disease recurrence can be successfully used to guide pre-emptive treatment. Platzbecker et al. evaluated the pre-emptive, MRD-triggered administration of AZA in two prospective studies. In RELAZA-1, a single-center phase II study of 20 patients with MDS/AML, AZA was administered pre-emptively after a decrease in CD34+ donor cell chimerism to <80% was detected. All patients received azacytidine for 4 cycles at a dose of 75 mg/m^2^ on day 1 to 7 resulting in an increase or stabilization of CD34+ DCC in 80% of patients. Hematological relapse eventually occurred in 65% of patients, but not until a median of 231 days after the initial decrease in CD34+ chimerism (40). Based on the encouraging results, a follow-up study was initiated (RELAZA2) (42). In this second prospective, multicenter trial, a cohort of 198 patients with AML or high-risk MDS were prospectively monitored using either CD34+ chimerism from the peripheral blood or disease specific markers such as mutant *NPM1*, or reciprocal translocations including *RUNX1::RUNX1T1, CBFb::MYH11* or *NUP214::DEK*. Patients (n=53) with AML or MDS becoming MRD-positive after transplantation (n=24) or after conventional chemotherapy (n=29) received standard dose azacitidine for 7 days monthly for up to 24 cycles. MRD positivity was defined by a decrease in donor CD34+ chimerism below 80% or an increase in mutant NPM1 or leukemia-specific fusion genes in the bone marrow or peripheral blood above 1%. RFS was 46% after one year and 26 (49%) patients eventually relapsed. The overall response rate (MRD negativity or MRD positivity without hematological relapse) in patients after allogeneic hematopoietic stem cell transplantation was 71% (17 of 24 patients). Treatment with azacytidine was not associated with increased myelotoxicity or aggravated GvHD. Interestingly, overall survival of MRD-positive patients who achieved a response with azacytidine was similar to that of MRD-negative patients. This observation suggests that the delay in disease recurrence achieved by a pre-emptive therapeutic intervention could be of considerable benefit for patients. The prolonged period of disease control may allow better recovery from early toxicities and more time for adequate scheduling of salvage therapy including first or second alloHCT. In a retrospective analysis performed by Sairafi and colleagues, 118 patients with hematologic malignancies were given DLI after alloHCT either because of hematologic relapse (n=44), molecular relapse based on leukemia lineage-specific chimerism analysis (n=52), or other causes (n=22). Patients with acute leukemia and MDS showed a significantly better 3-year OS of 42% if DLI treatment was given at the time of molecular relapse, compared to 16% at hematologic relapse (39). In a retrospective trial among 143 patients with AML and MDS having received alloHCT, Rosenow and colleagues reported that early DLI intervention based on diagnosis of incomplete CD34-lineage specific DCC can convert mixed DCC to complete DCC and thus prevent overt hematological relapse in 25 of 43 patients. Immune intervention consisted of rapid tapering of immunosuppressive treatment in 29 patients and/or DLI infusions in 10 patients (27). The benefit of MRD-triggered intervention with DLI in post-transplant AML was confirmed in two additional prospective trials (47, 48).

The combination of AZA and DLI is another promising concept of MRD-guided post-transplant intervention since it combines cytotoxic effects with an increase in allo-immune response and has already been proven feasible and safe in the salvage situation after allo-SCT (7). Guillaume and colleagues analyzed 77 patients (54 with AML, 23 with MDS) who had received at least 1 cycle of prophylactic or pre-emptive low-dose AZA with or without escalating doses of DLI following alloHCT (43). Among these patients, it was retrospectively determined that AZA/DLI was administered pre-emptively in 8 patients and prophylactically in 22 patients, depending on the presence or absence of MRD. In this retrospective evaluation, a 2-year OS of 70.8% and a 2-year progression-free survival (PFS) of 68% were observed with no significant difference in OS between the pre-emptive and prophylactic subgroups. Another option might be the combination with interferon-alpha, which might enhance the anti-leukemic activity and has been shown to be active when given as single agent post alloHCT in patients with impeding MRD [reviewed in (49)].

Taken together, these promising results clearly indicate that detection of MRD based on lineage-specific chimerism, in particular in circulating CD34+ cells, is feasible and allows for effective intervention, preventing or least substantially delaying hematological relapse and increasing disease free and overall survival. However, due to fact that many of these patients still relapse, further work is necessary to eliminate leukemia-initiating cells and to enable long term disease control and cure.

## Current Limitations and Future Developments

Although CD34 is expressed and therefor usable for enrichment of circulating blasts in about 70-80% of patients with AML, about 20-30% lack expression of this antigen (34). In these patients, the cKIT-receptor protein (CD117) might be an alternative, since it is also expressed on early normal hematopoietic cells as well as on 50-60% of leukemic blasts, about 10-15% of which lack CD34 expression. For these CD34-negative patients, CD117 might represent a useful antigen to select circulating leukemic cells in order to detect MRD. First data generated in the RELAZA2 study support a similar level of sensitivity (42).

NGS-based techniques are increasingly used for MRD detection (50). Using sophisticated molecular barcoding strategies combined with bioinformatic analysis, such as universal molecular identifiers (Heuser et al.), several groups were able to push the limits of detection of this technique down to 10^-4^ to 10^-6^ and show predictive potential in retrospective cohorts (8). However, although principally feasible, broad clinical application of this technique is currently still hampered by the considerable costs. Aguirre-Ruiz and coworkers recently performed a retrospective correlation of the results of NGS-MRD (level of sensitivity 10^-3^) and sensitive chimerism analysis using a commercial qPCR-assay in a pilot study of 20 patients with MDS or AML post alloHCT (51). The authors could document on overall concordance of the data, especially in patients with mixed chimerism, the detection of leukemic SNVs increased the predictive value of the chimerism findings. We recently explored an alternative approach, aiming at a combination of sorting circulating leukemic stem cells and targeted, ultradeep, error-controlled NGS. First validation experiments clearly support the feasibility of this approach, with an achievable sensitivity down to 1x10^-6^ (**Figure 1B**; Stasik et al., in revision). In addition, this approach might not only be useful in the post-transplant setting, but could potentially also facilitate sensitive MRD detection in patients after conventional treatment.

## Author Contributions

J-AG and CT wrote the manuscript. MB, SS, and UP read and edited the manuscript. All authors contributed to the article and approved the submitted version.

## Conflict of Interest

CT is co-owner and CEO of AgenDix GmbH, a company performing molecular diagnostics.

The remaining authors declare that the research was conducted in the absence of any commercial or financial relationships that could be construed as a potential conflict of interest.

## Publisher’s Note

All claims expressed in this article are solely those of the authors and do not necessarily represent those of their affiliated organizations, or those of the publisher, the editors and the reviewers. Any product that may be evaluated in this article, or claim that may be made by its manufacturer, is not guaranteed or endorsed by the publisher.
